# Comparability of accelerometry outcomes across popular metrics and widespread sensor positions

**DOI:** 10.1371/journal.pone.0337897

**Published:** 2025-12-03

**Authors:** Robin Olfermann, Ulrich Ebner-Priemer, Markus Reichert, Marco Giurgiu

**Affiliations:** 1 Junior Research Group eHealth and Sports Analytics, Faculty of Sport Science, Ruhr University Bochum, Bochum, North Rhine-Westphalia, Germany; 2 Department of Sport and Exercise Science, Research Group Sport and Exercise Psychology, Paris Lodron University Salzburg, Salzburg, Austria; 3 Mental mHealth Lab, Institute of Sports and Sports Science, Karlsruhe Institute of Technology, Karlsruhe, Baden-Wuerttemberg, Germany; 4 Department of Psychiatry and Psychotherapy, Central Institute of Mental Health, Medical Faculty Mannheim, Heidelberg University, Mannheim, Baden-Wuerttemberg, Germany; ASPIRE Academy for Sports Excellence, QATAR

## Abstract

Accelerometry is a state-of-the-art procedure to capture physical activity. However, the large variety of accelerometry metrics and wearing positions hamper the comparability of outcomes. Since this is a well-known challenge, we investigated how outcomes can be translated across four metrics and six sensor positions. Twenty healthy adults conducted 32 structured and semi-structured activities while wearing accelerometers at the hip, chest, thigh, wrist, ankle, and upper arm. The raw data was converted into four common metrics: Movement Acceleration Intensity (MAI), Euclidean Norm Minus One (ENMO), Mean Amplitude Deviation (MAD) and counts per minute (CPM), as computed by the Actigraph GT3X+ algorithm. Relationships between acceleration metrics and sensor positions were quantified via Pearson correlations and scatterplots. Our results show that nearby sensor positions were highly correlated (e.g., MAD hip and thigh: *r* = .96), while correlations between more distant sensor positions were weaker and less linear (e.g., MAD wrist and thigh: *r* = .80). Correlations between MAI, MAD and ENMO were high (*r* = .9), while correlations between CPM and other metrics were substantially lower (*r* = .78), less linear, and influenced by activity type. Thus, linear conversion between MAI, ENMO and MAD are highly feasible, but converting CPM may be less accurate. Linear conversions between nearby sensor positions are accurate, yet linear conversions between more distant sensor positions appear challenging. Importantly, based on 32 activities as well as metric- and sensor-location-specific configurations, we provide a comprehensive overview of outcome measures that enables researchers to individually explore conversion opportunities towards their own data.

## Introduction

Physical Activity (PA) in daily life is connected to numerous physical and mental health benefits [1, 2], whereas physical inactivity is recognized as a major risk factor for non-communicable diseases worldwide [3]. In order to better understand the relationship between PA and health outcomes it is important to accurately measure PA. While past research primarily relied on self-reports, nowadays researchers increasingly use wearables with built-in accelerometers to quantify PA via device-based measurements. Wearables enable continuous data collection in everyday life and offer advantages such as high temporal resolution, higher ecological validity, minimized recall bias, and the ability to quantify PA intensity [4]. Due to their scientific and commercial popularity, there are now a variety of different manufacturers and models on the market [5]. However, this variety led to a large heterogeneity of preprocessing algorithms for the raw acceleration data, which is reflected in various different metrics, such as Euclidean Norm Minus One (ENMO), Mean Amplitude Deviation (MAD), Movement Acceleration Intensity (MAI) or activity counts. Moreover, ActiGraph counts, a metric mainly established in the context of ActiGraph devices, differ between model generations and are based on different preprocessing algorithms [6].

In addition to the large heterogeneity of algorithms and metrics, there is no standardized way of using these devices. For example, despite years of using accelerometers, there is no consensus on where to wear them [7]. Commonly, sensors are worn on the waist, wrist, chest or on the thigh [7, 8]. Depending on the research question and device, different wearing positions come with advantages and disadvantages. For example, sensors at the wrist may minimize participant burden and increase adherence [7] but may overestimate daily physical activity because of upper extremity movement, such as drawing or writing [9], which involve relatively low energy expenditure compared to movements of the lower limbs. Some wearables placed at the wrist or chest are able to measure heart rate [10] while some wearables attached to the thigh are able to detect whether the subject is sitting or lying down based on the sensor orientation [11]. The wearing position therefore often depends on the nature of the research question. However, wearing sensors at different parts of the body greatly influences the acceleration data, which has been extensively demonstrated [7, 12]. To illustrate, a wrist-worn accelerometer naturally senses much less movement when cycling than a sensor placed on the thigh. Therefore, especially in free-living studies, in which it is uncommon to know what activity was performed, it is difficult to compare acceleration data from different wearing positions. Thus, comparability between studies is limited due to the heterogeneity of the used accelerometers and the wearing protocols. The ability to convert from metric to metric and between wearing positions would improve comparability.

To date, only few studies have systematically compared metrics across limited sensor positions. For instance, laboratory-based comparisons computed ENMO and MAD intensity-based thresholds distinguishing sedentary behavior from light physical activity for wrist- and hip-worn devices [13]. Free-living comparisons have shown systematically higher acceleration values at the dominant compared to the non-dominant wrist, with ENMO, low-pass filtered ENMO (LFENMO), and MAD being highly comparable across wrist placements and more consistent than ActiGraph GT3X+ counts [14]. However, to our knowledge, no study has systematically compared both multiple accelerometer metrics and several sensor locations under laboratory conditions, with a structured activity protocol.

Therefore, we conducted a comprehensive comparison of four metrics and six sensor locations across 32 structured and semi-structured activity conditions. Metrics we aim to compare are ENMO, MAD, MAI and counts per minute (CPM), as computed by the Actigraph GT3X+ algorithm. Sensor locations we aim to compare are wrist, hip, thigh, chest, ankle and upper arm. Specifically, the aim of this study is twofold: First, to provide a comprehensive, descriptive overview of the chosen metrics, across a wide range of activities and sensor positions, and second, to explore potential linear associations between accelerometry metrics and between wearing locations for future conversion.

## Materials and methods

### Participants

Data used in this study were collected as part of a previous study to validate accelerometers for the assessment of body postures and sedentary behavior [15]. The study included a convenience sample of 20 healthy adults consisting of staff and students of the Karlsruhe Institute of Technology (KIT), recruited between 01.08.2018 and 31.09.2018. The study was approved by the ethics committee at the KIT. Prior to obtaining written informed consent, all eligible participants were provided with both written and verbal information on the study procedures. Participants had the opportunity to withdraw from the study at any point in time.

### Procedure

During the study, participants followed a structured protocol consisting of 32 consecutive conditions, each performed for two to five minutes. The protocol was divided into two sections: a fully standardized section (e.g.,: lying horizontal, sitting leaned backwards etc.) and a semi-standardized section (e.g., tidying up, walking 3.2 km/h, cycling etc.; for a full overview of all conditions see S1 Table, Supporting Information 1). All conditions were conducted in a laboratory setting, which included a gym and an outdoor area (400 m all-weather running track). To ensure that participants maintained the same constant speed during the walking and running conditions, a research assistant used a bicycle equipped with a speedometer, walking beside participants during the walking trials and cycling alongside them during the running trials. To ensure synchronization of the activity monitors, all monitors were initialized on the same computer prior to testing, and participants performed three vertical jumps at the beginning and end of the measurement. Participants’ height and weight were measured using an electronic column scale (seca GmbH & Co. KG, Hamburg, Germany) without shoes before testing. Six Move 4 (movisens GmbH, Karlsruhe, Germany) activity monitors were fitted to each participant at six different body positions before testing and worn concurrently throughout the entire study period.

### Activity monitors

Move 4 activity monitors were placed on the chest (using disposable electrodes), on the top of the anterior superior iliac spine on the right side of the hip (with an elastic belt), on the lateral part of the right thigh (using TegadermTM skin tape), on the lateral aspect of the right ankle (using an ankle band), on the lateral aspect of the dominant upper arm (using an upper arm band) and on the dominant wrist (using a wrist band). These devices, measuring 62 × 39 × 11 mm and weighing 25 g, are capable of recording triaxial acceleration at a range of ±16 g with a sampling rate of 64 Hz. The acceleration sensors had been calibrated by the manufacturer for all axes (offset and sensitivity). The manufacturer’s software, SensorManager (version 1.11.19), was used to initialize and download the data, while their software DataAnalyzer (version 1.13.5) was used to calculate data with a one-second resolution. We chose a one-second resolution to capture the activities as accurately as possible, each of which lasted between 2 and 5 minutes. The following four accelerometer metrics were calculated for each sensor location separately: MAI, MAD, ENMO and ActiGraph GT3X + CPM.

### Data processing

MAI is calculated by bandpass filtering the raw signals of the three accelerometer axes (Butterworth 0.25–11 Hz, 4th order), to ensure that signals not produced by PA are removed. Afterwards the Euclidean norm of the three axes is calculated and averaged over the given interval. The output is then expressed in mg [16].

ENMO is based on the Euclidean norm of the three accelerometer axes, with the gravitational acceleration of 1g being subtracted from it afterwards. To prevent negative results from subtraction, values that are less than zero are set to zero. All values within a given interval are then averaged and expressed in mg as the final output [17].

MAD is also based on the Euclidean norm of the three accelerometer axes. The values initially comprise the tri-axial dynamic component caused by deviations in velocity and the static gravitational component. After the static component is removed from the interval, the remaining values (the dynamic component) are revised. All revised values within the given interval are then averaged and expressed in mg as the final output [18].

ActiGraph GT3X+ counts are first bandpass filtered around frequencies compatible with human activity and then quantized by a 12-bit analog-to-digital converter, where each level beyond 128 is considered to be 1 count [6]. These counts were then summed within 1 second epochs and upscaled by averaging per condition and multiplying by 60 to obtain counts per minute (CPM). The three axes were combined into vector magnitude by square rooting the sum of the squares of each axis of data.

### Statistical analyses

Before the main analysis, we excluded the first and last five seconds per activity to eliminate possible biases (e.g., when starting or ending an activity). We ensured that all participants had the same length of measurements per condition by truncating any additional seconds (participants usually performed the conditions a few seconds longer to ensure that the required minimum measuring length has been obtained). For overview purposes, we then grouped all 32 conditions into the following eight superordinate categories: lying, sitting, standing, activities of daily living (adl), climbing stairs, walking, jogging and cycling. We then first calculated descriptive statistics (means and standard deviations) for all eight activity categories per sensor location and per metric (for descriptives of all 32 conditions, see S2 Table, Supporting Information 2). Second, we investigated and visualized between-person variance using standard deviations and boxplots. Third, we calculated Pearson correlation coefficients between all metrics and all sensor locations using data at the temporal resolution of one second. Fourth, we visualized relationships between every pairwise combination of the four metrics and the six sensor locations using scatterplots. As an additional analysis we further calibrated metric- and sensor-location-specific cut-points for different physical activity intensity zones (detailed information on methods and results of the cut-points-analysis are provided in S3 File). Data wrangling and statistical analyses were performed with R. All data and analysis code are openly available on the Open Science Framework (OSF) at https://doi.org/10.17605/OSF.IO/JQWYT.

## Results

Data from 20 healthy participants were included in our final analyses. The sample consisted of ten men and ten women, ranging in age from 18 to 32 years (*M* = 25.68, *SD* = 4.55) and with a BMI of 18.28 to 30.93 kg/m^2^ (*M* = 22.90, *SD* = 3.43). In total, we analyzed 103549 seconds of accelerometry data (i.e., on average 86 minutes and 17.45 seconds per participant).

### Descriptives across all metrics and sensor locations

Acceleration values vary across activity categories, metrics and sensor location. Table 1 provides a comprehensive overview of metric- and sensor-location-specific means and standard deviations for all eight activity categories (lying, sitting, standing, activities of daily living, climbing stairs, walking, jogging, cycling). The rows of Table 1 display the activity categories grouped by sensor location (thigh, hip, chest, ankle, wrist, upper arm). The columns of Table 1 indicate the metric (MAI, ENMO, MAD and CPM). The table demonstrates four issues: First, average acceleration values differ between sensor positions. For example, when comparing the values from the first block (thigh) with those from the fifth block (wrist), it is noticeable that for many activities the average wrist values are more than twice as high than the average thigh values. Second, the ratios between activities differ between sensor positions: The ratio of jogging and cycling measured at the thigh (CPM) is 2.3 (12986/5634), while the ratio for jogging and cycling measured at the hip (CPM) is 8.7 (8928/1021). Third, average acceleration values differ between metrics: For example, cycling measured at the thigh has a mean of 99 expressed in ENMO and a mean of 158 expressed in MAD. Fourth, the ratios between activities differ between metrics: The ratio of jogging and cycling for CPM (thigh) is 2.3 (12986/5634), while the ratio for jogging and cycling for ENMO (thigh) is 9.5 (936/99). Thus, comparing mean acceleration data and their activity-specific relations across sensor positions or metrics can be highly inaccurate. A detailed overview of mean values for all 32 activities is provided in S2 Table in Supporting Information 2 and online via an interactive web application [19].

**Table 1:** Mean acceleration values.

location	category	MAI	ENMO	MAD	CPM
thigh	lying	6 (31)	11 (13)	2 (11)	59 (703)
	sitting	9 (20)	7 (7)	4 (8)	26 (321)
	standing	56 (64)	12 (14)	13 (17)	404 (892)
	adl	132 (131)	35 (50)	48 (59)	1812 (2230)
	climbing stairs	467 (157)	208 (107)	308 (98)	6119 (2076)
	walking	466 (182)	228 (141)	293 (159)	4740 (1529)
	jogging	1295 (326)	936 (362)	839 (242)	12986 (3992)
	cycling	259 (107)	99 (65)	158 (82)	5634 (2961)
hip	lying	6 (23)	8 (8)	2 (8)	41 (542)
	sitting	8 (13)	13 (7)	3 (3)	12 (202)
	standing	51 (60)	21 (18)	18 (24)	271 (672)
	adl	103 (94)	32 (28)	39 (42)	1365 (2022)
	climbing stairs	353 (136)	145 (78)	254 (103)	4944 (1975)
	walking	304 (104)	118 (53)	193 (83)	3200 (1263)
	jogging	1005 (231)	519 (152)	745 (159)	8928 (2000)
	cycling	125 (53)	51 (25)	69 (29)	1021 (1152)
chest	lying	8 (26)	8 (8)	4 (8)	51 (632)
	sitting	15 (9)	19 (8)	7 (6)	32 (362)
	standing	70 (78)	30 (21)	28 (33)	560 (1308)
	adl	119 (102)	34 (25)	42 (38)	1945 (2930)
	climbing stairs	332 (127)	146 (76)	259 (102)	5242 (2152)
	walking	248 (92)	105 (45)	174 (73)	2787 (1569)
	jogging	983 (242)	501 (150)	794 (191)	10301 (2331)
	cycling	113 (53)	50 (24)	66 (28)	441 (774)
ankle	lying	6 (34)	12 (17)	3 (15)	67 (679)
	sitting	10 (31)	6 (12)	4 (12)	49 (515)
	standing	31 (39)	9 (13)	9 (13)	204 (649)
	adl	150 (176)	63 (108)	80 (113)	2229 (3121)
	climbing stairs	678 (245)	420 (217)	525 (150)	11479 (3768)
	walking	626 (219)	446 (212)	511 (194)	10062 (4029)
	jogging	1438 (351)	1330 (484)	1082 (238)	28539 (7072)
	cycling	387 (208)	175 (123)	258 (136)	9728 (6037)
wrist	lying	11 (55)	9 (17)	4 (17)	158 (1318)
	sitting	44 (85)	10 (19)	14 (24)	607 (2112)
	standing	336 (481)	164 (291)	200 (300)	6382 (9775)
	adl	339 (273)	99 (118)	142 (120)	7292 (6912)
	climbing stairs	398 (172)	160 (93)	253 (103)	7469 (3849)
	walking	286 (151)	138 (103)	171 (76)	4983 (4111)
	jogging	1479 (556)	841 (358)	891 (332)	22902 (8436)
	cycling	167 (100)	80 (52)	106 (46)	1435 (2426)
upper arm	lying	9 (40)	10 (14)	3 (12)	101 (952)
	sitting	24 (38)	9 (10)	9 (11)	143 (750)
	standing	179 (251)	73 (130)	94 (148)	2312 (3761)
	adl	210 (161)	55 (58)	80 (67)	3914 (4085)
	climbing stairs	370 (142)	143 (82)	247 (104)	6206 (2441)
	walking	249 (85)	91 (43)	149 (56)	3098 (1872)
	jogging	1212 (400)	652 (276)	724 (224)	17765 (6689)
	cycling	196 (69)	74 (34)	108 (36)	1796 (1741)

Standard deviations are shown in brackets. Adl = activities of daily living.

### Descriptives on a person level across all metrics and sensor locations

**Fig 1:**
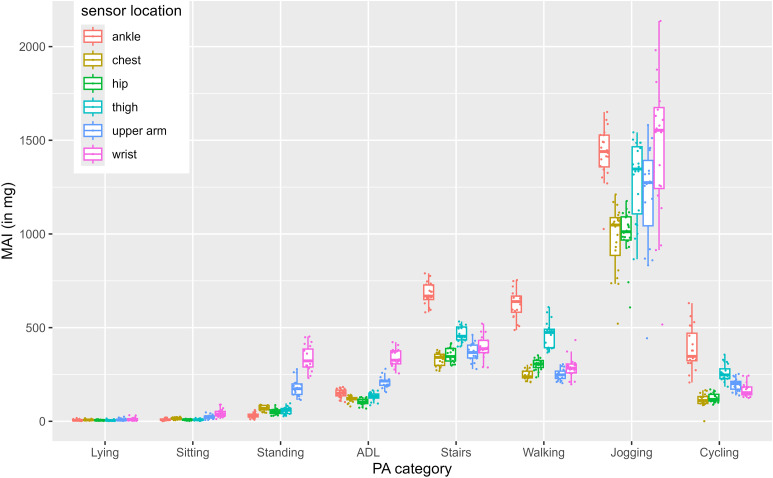
MAI data across sensor locations and activity categories. Activities are shown on the x-axis, grouped by sensor location. The y-axis displays the participant means of the respective metric. Shorter boxplots indicate smaller between-person variance while taller boxplots indicate larger between-person variance. Adl = activities of daily living.

**Fig 2:**
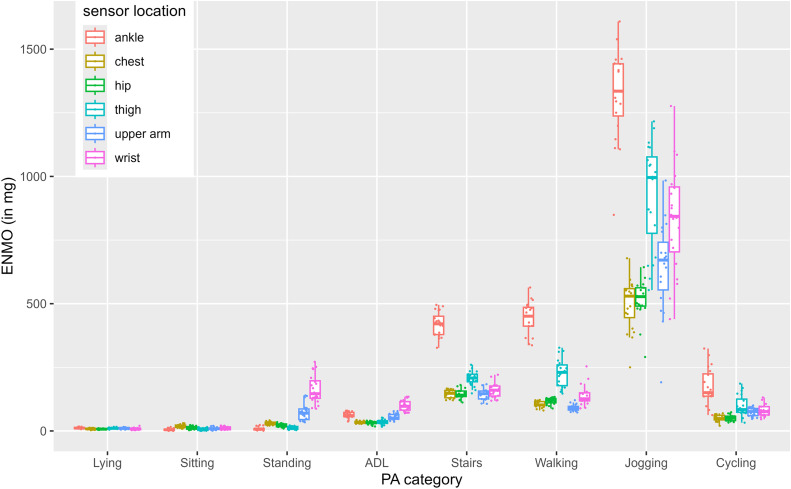
ENMO data across sensor locations and activity categories.

**Fig 3:**
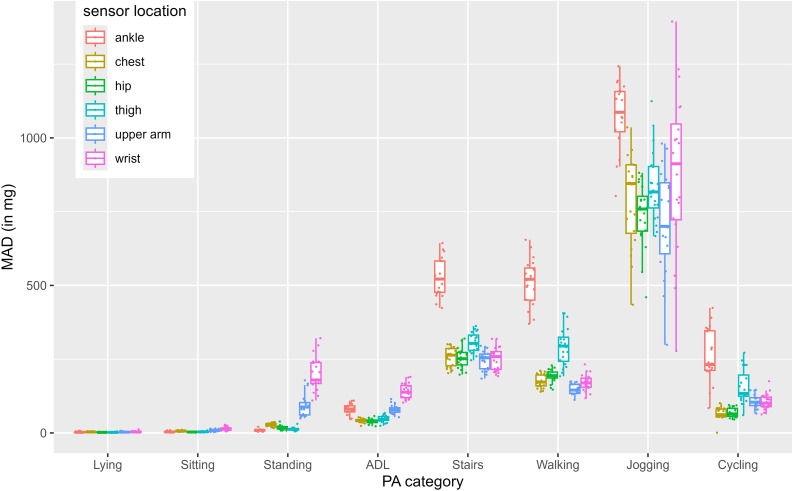
MAD data across sensor locations and activity categories.

**Fig 4:**
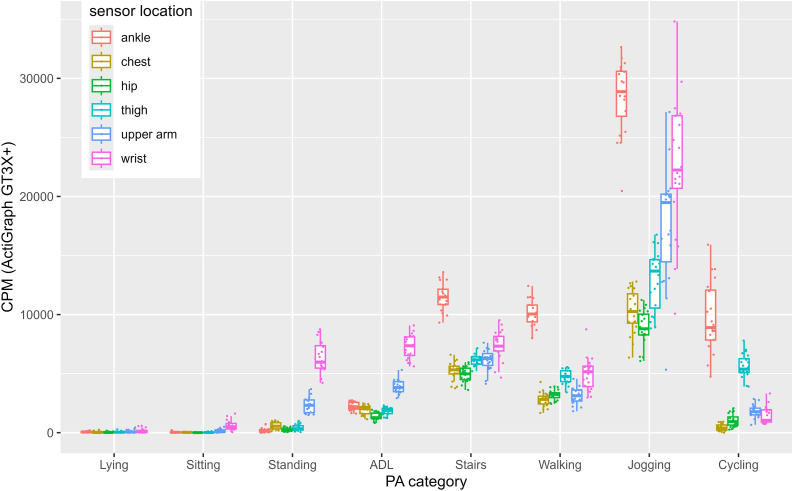
CPM data across sensor locations and activity categories.

In our second part, we visualized the variance between participants for each metric across all sensor locations and activity categories (see Figs 1–4). For example, all 4 Figs show that wrist-sensors measure more variance between people when jogging than sensors at other locations.

Standard deviations of the participant means within the MAI metric ranged from *SD* = 2.81 mg (lying measured with hip-worn sensors) to *SD* = 393.25 mg (jogging measured with wrist-worn sensors). Standard deviations of the participant means within the ENMO metric ranged from *SD* = 2.11 mg (lying measured with hip-worn sensors) to *SD* = 209.49 mg (jogging measured with wrist-worn sensors). Standard deviations of the participant means within the MAD metric ranged from *SD* = 0.97 mg (sitting measured with hip-worn sensors) to *SD* = 274.82 mg (jogging measured with wrist-worn sensors). Standard deviations of the participant means within the counts metric ranged from *SD* = 13.96 CPM (sitting measured with hip-worn sensors) to *SD* = 5775.11 CPM (jogging measured with wrist-worn sensors).

For all four metrics, hip worn-sensors show the smallest between-person variance with average standard deviations of *SD* = 32.61 for MAI, *SD* = 17.63 for ENMO, *SD* = 24.56 for MAD and *SD* = 1498.57 for CPM, whereas wrist-worn sensors show the highest between person variance with average standard deviations of *SD* = 85.39 for MAI, *SD* = 48.52 for ENMO, *SD* = 57.46 for MAD, and *SD* = 1498.57 for CPM.

### Correlations across all metrics and sensor locations

Linear relationships between sensor locations and metrics were analyzed by calculating the bivariate Pearson correlation coefficients on the level of seconds (displayed in Table 2).

**Table 2:** Correlations between metrics and sensor locations.

		MAI						ENMO						MAD						CPM					
		ankle	chest	hip	thigh	arm	wrist	ankle	chest	hip	thigh	arm	wrist	ankle	chest	hip	thigh	arm	wrist	ankle	chest	hip	thigh	arm	wrist
**MAI**	ankle	–																							
	chest	.91	–																						
	hip	.95	.98	–																					
	thigh	.97	.95	.98	–																				
	arm	.83	.94	.92	.89	–																			
	wrist	.71	.86	.82	.78	.95	–																		
**ENMO**	ankle	.97	.92	.94	.96	.84	.73	–																	
	chest	.89	.97	.95	.93	.91	.83	.92	–																
	hip	.91	.95	.98	.95	.90	.81	.94	.97	–															
	thigh	.92	.93	.95	.97	.88	.78	.96	.95	.96	–														
	arm	.80	.90	.88	.86	.96	.86	.84	.90	.89	.88	–													
	wrist	.75	.87	.84	.81	.92	.94	.78	.87	.85	.83	.89	–												
**MAD**	ankle	.97	.89	.93	.95	.80	.68	.95	.88	.90	.91	.78	.73	–											
	chest	.91	.97	.97	.95	.91	.83	.93	.98	.97	.95	.90	.86	.91	–										
	hip	.93	.96	.98	.96	.90	.80	.94	.97	.98	.95	.88	.84	.93	.99	–									
	thigh	.95	.93	.95	.97	.85	.75	.96	.93	.95	.96	.84	.80	.96	.94	.96	–								
	arm	.85	.93	.92	.90	.96	.87	.86	.92	.91	.89	.94	.87	.85	.94	.92	.88	–							
	wrist	.76	.89	.86	.83	.94	.95	.78	.87	.85	.83	.88	.93	.74	.88	.85	.80	.93	–						
**CPM**	ankle	.98	.91	.94	.96	.84	.72	.95	.91	.92	.92	.82	.77	.95	.92	.93	.95	.87	.77	–					
	chest	.78	.90	.86	.82	.83	.76	.76	.81	.80	.78	.75	.74	.76	.83	.82	.79	.80	.77	.77	–				
	hip	.86	.90	.92	.89	.82	.73	.83	.85	.86	.83	.76	.73	.84	.87	.88	.86	.82	.76	.85	.92	–			
	thigh	.92	.89	.91	.94	.83	.73	.88	.86	.87	.88	.80	.75	.89	.87	.88	.90	.84	.77	.94	.81	.88	–		
	arm	.74	.86	.82	.80	.95	.88	.74	.81	.80	.78	.89	.82	.70	.81	.80	.76	.88	.85	.75	.81	.77	.77	–	
	wrist	.59	.74	.69	.65	.85	.94	.60	.68	.67	.64	.72	.84	.55	.68	.66	.61	.74	.84	.59	.70	.65	.62	.84	–

**Fig 5:**
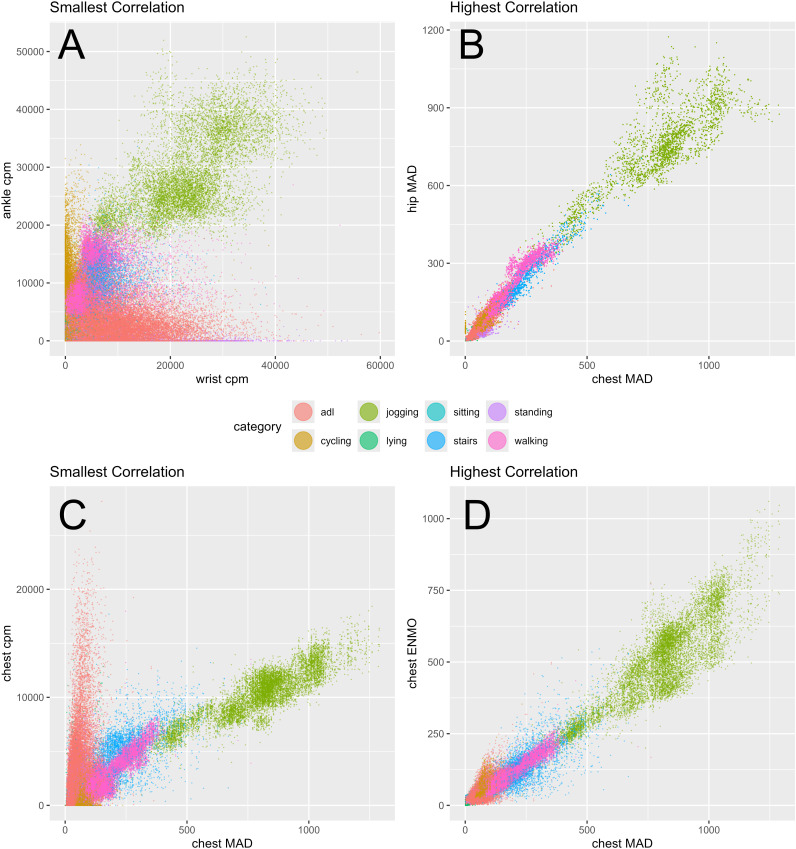
Smallest and highest correlations between sensor locations and between metrics. Panel A: smallest correlation between the same metrics recorded at different sensor locations. Panel B: highest correlation between the same metrics recorded at different sensor locations. Panel C: smallest correlation between different metrics recorded at the same sensor location. Panel D: highest correlation between different metrics recorded at the same sensor location. Acceleration values are colored according to activity category. Adl = activities of daily living.

When looking at relationships between sensor positions, it is visible that the strength of the correlations differs between the metrics. The average correlation coefficient between sensor locations is larger for the metrics MAI, ENMO, and MAD (r = .9) than for CPM (r = .78). The smallest correlation coefficient between different positions within the same metric is found for data measured in CPM between wrist-worn sensors and ankle-worn sensors (r = .59), see Fig 5 panel A. The highest correlation coefficient between different locations within the same metric is found for MAD data between chest-worn sensors and hip-worn sensors (r = 0.99), see Fig 5 panel B. While the relationship between chest and hip sensors for MAD data is linear and similar for all activities, the relationship between thigh and wrist sensors for CPM data is smaller, less linear and differs between activities.

When looking at relationships between metrics, the smallest correlation coefficient between different metrics measured at the same sensor position is found for chest-measured data between MAD and CPM (r = .83), see Fig 5 panel C. The highest correlation coefficient between different metrics for the same sensor position is found for chest-measured data between ENMO and MAD (r = .98), see Fig 5 panel D. While the relationship between MAD and ENMO is linear and similar for all activities, the relationship between MAD and CPM is smaller, less linear and differs between activities. Especially the relationship between MAD and CPM for activities of daily living (red) differs from other activities. Visualizations of all 276 pairwise relationships as well as their regression models are provided online via an interactive web application [19].

## Discussion

Nowadays, many researchers use wearables with built-in accelerometers to measure PA. However, there is neither a standardized way of using these devices nor a uniform metric to report the acceleration data. This makes comparability between studies difficult. Therefore, in a comprehensive descriptive overview, we first have shown how accelerometer data vary between four different metrics, six different sensor positions, and 32 activities across and between persons. Second, we explored conversion opportunities between accelerometry metrics and between data from different sensor locations. We show that data collected from different wearing positions and measured in different metrics are highly linear correlated but the strength of the correlation depends on the activity, metric and sensor location.

Table 1 provides a detailed overview of average accelerometer data across 32 different activities. This overview provides a reference point for future studies, enabling comparisons between different activity data depending on the metric and sensor position used. Table 1 displays some common accelerometry phenomena. For instance, it is known that wrist-placed accelerometers may overestimate time in lowest activity intensities (e.g., sedentary behavior) that involves upper extremity movement [9] and that wrist-placed sensors are incapable of measuring nuanced movements when cycling. From our data, we can see that wrist-worn sensors measure relatively small means during cycling (MAI: 167 mg, ENMO: 80 mg, MAD 106 mg, CPM: 1435), especially compared to most activities of daily living that usually involve more upper extremity movement such as hanging out laundry (MAI: 453 mg, ENMO: 113 mg, MAD 178 mg, CPM: 5326). Even sedentary activities such as reading the newspaper (MAI: 130 mg, ENMO: 22 mg, MAD 36 mg, CPM: 2562) can have quite high mean values when measured with wrist-worn sensors. Our results are broadly comparable to previous findings. For example, the mean ENMO values for adl were 32 mg at the hip and 99 mg at the wrist, which are comparable to those reported by Bakrania et al. [13] for various household activities (5.9–25.8 mg for hip and 53.8–118 mg for wrist, depending on activity and sensor). Similarly, the mean MAD values detected for adl (39 mg for hip, 142 mg for wrist) align well with their reported ranges (6.9–44.2 mg for hip and 84.2–174.3 mg for wrist). Overall, our results are consistent with prior studies in showing higher acceleration magnitudes for wrist- compared to hip-worn sensors, particularly for adl, and higher values for MAD compared to ENMO [13].

With regard to the comparability of accelerometer data, Table 1 demonstrates four core issues: First, average acceleration values differ between sensor positions. Second, the ratios between activities differ between sensor positions. Third, average acceleration values differ between metrics. Fourth, the relations between activities differ between metrics. Thus, not only do the average acceleration values differ between metrics and between sensor positions, but the relations between different activities are also influenced by the sensor position and the metric. Consequently, a simple comparison of mean acceleration data and their activity-specific relations across different metrics or sensor positions is inaccurate.

Data presented in Figs 1-4 reveal numerous aspects that merit further discussion. In particular, across all metrics, wrist-placed sensors measure the highest between person variance when jogging (MAI: *SD* = 393.25 mg, ENMO: *SD* = 209.49 mg, MAD: *SD* = 274.82 mg, CPM: *SD* = 5775.11). Although all subjects ran at the same speed, there are strong differences between the mean values of the subjects when measured with the sensors attached to the wrist. This difference is most likely explained by individual differences in arm swing during running. For example, participants with a more pronounced arm swing may display higher acceleration values at the same running speed than those with a more restrained arm movement. Thus, inferring energy expenditure solely from aggregated wrist acceleration data can be misleading, as they conflate arm swing and lower-limb activity. Notably, arm swing itself has been associated with reduced metabolic cost during running [20, 21]. Frequency-domain features from raw data may help disentangle these components. These findings illustrate that acceleration data are greatly influenced by sensor position. While sensor placement was carefully standardized and controlled in this study, inconsistencies in fit or attachment could also increase between-subject variability. In observational, interventional or surveillance studies, a single accelerometer is typically used. This keeps costs and data processing effort low and compliance high. However, as sensors are getting smaller and cheaper, researchers may consider using more than one sensor attached at multiple locations to improve activity detection and energy expenditure estimations or broaden the number of extracted data features. In 2020, Tang et al. demonstrated that posture is best detected with at least two non-wrist sensors and PA is best recognized with a combination of at least one wrist and one non-wrist sensor [22]. Moreover, combining accelerometers from multiple body locations can substantially improve activity type recognition. Identifying specific activity types based on accelerometer data has long been an ambitious goal in PA research [23]. Machine learning approaches have shown particular promise in this area. While reliable detection of complex daily activities remains challenging, basic activity types such as walking, running, cycling, or stair climbing can be recognized more consistently. Especially when data from multiple sensors are integrated, classification performance improves. For example, approaches such as Kalman filtering when combining accelerometer and gyroscope data, or posterior-adapted class-based weighted decision fusion, have been shown to outperform traditional single-sensor methods [24, 25].

Another characteristic also becomes apparent when comparing Figs 1-4. That is, MAD jogging data, does not vary as much between sensor locations as jogging data of the other three metrics. Jogging data expressed in CPM seems to differ the strongest between sensor locations (e.g., ankle, wrist, chest, hip and thigh have very different means). This suggests that comparability and convertibility between wearing positions might depend on the metric.

To enable better comparability between study results, it would be practical to convert between metrics and between sensor positions. Therefore, we explored the linear relationship between metrics and between sensor positions with correlations and scatter plots. On this basis, we conclude that it is possible to convert linearly between MAI, ENMO and MAD because they generally have high linear relationships with one another (see Table 2 and Fig 5, panel D). However, in line with previous research demonstrating stronger associations among unfiltered metrics (ENMO, LFENMO, MAD) than with Actigraph GT3X+ counts [14] we found correlations between CPM and other metrics to be substantially lower, less linear, and more influenced by activity type (see for example Fig 5, panel C). Therefore, linear conversions between CPM and other metrics might be less accurate. Moreover, conversion between sensor positions seem also possible for some positions. While some relationships between positions are high and linear (e.g., chest and hip; see Fig 5, panel C), other relationships between positions are smaller and less linear (e.g., thigh and wrist; see Fig 5, panel A). Therefore, linear conversions between nearby sensor positions might be indeed possible and accurate. However, linear conversions between distant sensor positions are less accurate. In line with previous research [14], correlations between sensor positions were higher for MAI, ENMO, and MAD compared to CPM. However, unlike previous findings reporting higher consistency for MAD than for ENMO [14], our results showed no meaningful difference between these two metrics in terms of average correlations across the six sensor locations. In future, harmonization of metrics might be standard with specific software such as ActiPass [26], which processes raw accelerometry data from various sensors and outputs 24-hr cycle behavior constructs like different intensity zones or biological states such as being awake or sleeping [27]. Researchers started to identify the optimal 24-h pattern of PA for health with the aim to create public health recommendations. Rosenberger and colleagues therefore introduced the 24-h Activity Cycle as a new paradigm in health research [28].

While our study provides a comprehensive overview of descriptives and comparability measures between various accelerometry metrics and sensor positions, it is not free of limitations. First, the generalizability and comparability of our findings to other studies is limited by the use of a convenience sample of healthy adults, who may differ in fitness level from other demographics, and by possible differences in sensor brands and models. Different sensor models may introduce variations in raw accelerometer data, potentially leading to differences in derived metrics and outputs, thereby complicating comparability across studies [29]. A logical next step would be to replicate these comparisons across diverse other populations and sensor brands. Second, as our analyses focused exclusively on raw acceleration data rather than physiological measures (e.g., heart rate or oxygen consumption), results cannot be directly interpreted in terms of energy expenditure or physiological exercise intensity, a proposal that should be tackled by future studies. Third, the activities were not selected in a systematic way and might not be fully generalizable to everyday life. Fourth, we only investigated linear relationships between sensor positions and metrics. However, metrics and data from different sensor positions may still be interconvertible via nonlinear techniques such as certain machine learning methods.

## Conclusions

Our work can help researchers to better understand and compare accelerometry data, whether simply by providing metric- and sensor-position specific reference values for many activities or as a tool for linear conversion between metrics and between sensor placements. Based on our results, models can be selected that allow data to be converted between some specific metrics (e.g., between MAI, ENMO and MAD) or between some sensor positions (e.g., between hip-, chest- and thigh-worn sensors) to further increase comparability between studies.

## Supporting information

S1 Table Activity conditions. (DOCX)

S2 Table Means and standard deviation for all 32 conditions per metric and sensor location. (DOCX)

S3 File Cutpoint analysis. (DOCX)
