# Supplementary material for: Comparability of accelerometry outcomes across popular metrics and widespread sensor positions
Source: PLoS One. 2025 Dec 3;20(12):e0337897. doi: 10.1371/journal.pone.0337897 (PMC12674572; doi:10.1371/journal.pone.0337897)
Supplement: S1 Table — (DOCX) [file pone.0337897.s001.docx]

**Supporting information 1 – Activity Conditions**

**Table S1. Conditions Performed by the Participants**

| **Condition** | **Category** | **Duration**  (min) | **MET** | **Activity Intensity** |
| --- | --- | --- | --- | --- |
| 1. Natural lying | Lying | 2 | 1.0 |  |
| 1. Lying horizontal |  | 2 | 1.0 |  |
| 1. Lying left |  | 2 | 1.0 |  |
| 1. Lying right |  | 2 | 1.0 |  |
| 1. Lying prone |  | 2 | 1.0 |  |
| 1. Reclining | Sitting | 2 | 1.3 |  |
| 1. Natural sitting |  | 2 | 1.5 |  |
| 1. Sitting leaned forwards |  | 2 | 1.3 |  |
| 1. Sitting leaned backwards |  | 2 | 1.3 |  |
| 1. Sitting crossed legs right |  | 2 | 1.3 |  |
| 1. Sitting crossed legs left |  | 2 | 1.3 |  |
| 1. Natural standing | Standing | 2 | 2.5 | LPA |
| 1. Standing still |  | 2 | 1.3 |  |
| 1. Standing still (upper body movement) |  | 2 | 1.8 | LPA |
| 1. Light activity | Activities of daily life  (ADL) | 3 | 2.0 | LPA |
| 1. Working on a computer |  | 3 | 1.5 |  |
| 1. Set the table |  | 3 | 2.5 | LPA |
| 1. Reading newspaper |  | 3 | 1.3 |  |
| 1. Tidying up |  | 3 | 2.3 | LPA |
| 1. Get dressed |  | 3 | 2.5 | LPA |
| 1. Putting sheets on bed |  | 3 | 4.0 | MPA |
| 1. Using smartphone |  | 3 | 1.3 |  |
| 1. Hanging out laundry |  | 3 | 2.0 | LPA |
| 1. Vacuuming |  | 3 | 3.3 | MPA |
| 1. Magnetic board cleaning |  | 3 | 3.2 | MPA |
| 1. Climbing and descending stairs | Stairs | 3 | 4.0 | MPA |
| 1. Walking 2.8 km/h | Walking | 5 | 2.0 | LPA |
| 1. Walking 3.2 km/h |  | 5 | 2.8 | LPA |
| 1. Walking 5.4 km/h |  | 5 | 3.5 | MPA |
| 1. Jogging 7.6 km/h | Jogging | 5 | 8.3 | VPA |
| 1. Jogging 12.0 km/h |  | 3 | 11.8 | VPA |
| 1. Cycling | Cycling | 5 | 4.0 | MPA |
