# Supplementary material for: Comparability of accelerometry outcomes across popular metrics and widespread sensor positions
Source: PLoS One. 2025 Dec 3;20(12):e0337897. doi: 10.1371/journal.pone.0337897 (PMC12674572; doi:10.1371/journal.pone.0337897)
Supplement: S2 Table — (DOCX) [file pone.0337897.s002.docx]

**Supporting information 2 - Means and Standard Deviation for all 32 Conditions per Metric and Sensor Location**

**Table S2. Means and Standard Deviation for all 32 Conditions per Metric and Sensor Location**

| **location** | **category** | **condition** | **MAI** | **ENMO** | **MAD** | **CPM** |
| --- | --- | --- | --- | --- | --- | --- |
| thigh | lying | lying horizontal | 3 (2) | 6 (5) | 2 (1) | 0 (0) |
|  | lying | lying left | 4 (6) | 25 (7) | 2 (2) | 1 (37) |
|  | lying | lying prone | 9 (52) | 13 (17) | 4 (20) | 135 (1147) |
|  | lying | lying right | 1 (0) | 0 (1) | 1 (0) | 0 (0) |
|  | lying | natural lying | 12 (45) | 10 (13) | 4 (13) | 159 (1063) |
|  | sitting | natural sitting | 14 (37) | 9 (10) | 6 (14) | 104 (718) |
|  | sitting | reclining | 6 (13) | 8 (7) | 3 (6) | 11 (143) |
|  | sitting | sitting - crossed legs left | 7 (11) | 9 (7) | 3 (4) | 7 (108) |
|  | sitting | sitting - crossed legs right | 8 (10) | 4 (3) | 4 (4) | 6 (153) |
|  | sitting | sitting - leaned backward | 8 (15) | 8 (7) | 4 (6) | 15 (145) |
|  | sitting | sitting - leaned forward | 11 (17) | 8 (7) | 4 (8) | 14 (136) |
|  | standing | Standing still | 11 (13) | 6 (5) | 3 (3) | 12 (138) |
|  | standing | natural standing | 39 (40) | 8 (8) | 8 (9) | 319 (846) |
|  | standing | standing still – upper movement | 118 (65) | 22 (19) | 28 (20) | 880 (1125) |
|  | adl | Windows cleaning | 148 (79) | 26 (27) | 35 (24) | 2190 (1744) |
|  | adl | Setting table | 119 (100) | 25 (35) | 35 (36) | 1531 (1911) |
|  | adl | get dressed | 155 (125) | 38 (48) | 56 (54) | 2116 (2788) |
|  | adl | hang out the laundry | 152 (109) | 33 (43) | 46 (43) | 2177 (2190) |
|  | adl | light activity | 334 (110) | 122 (63) | 165 (61) | 4210 (1477) |
|  | adl | put clean sheets on the bed | 166 (129) | 42 (57) | 57 (60) | 2155 (2103) |
|  | adl | reading newspaper | 13 (28) | 8 (11) | 5 (12) | 44 (315) |
|  | adl | smartphone usage | 70 (121) | 21 (36) | 26 (46) | 1081 (2298) |
|  | adl | tidy up | 98 (109) | 25 (37) | 35 (42) | 1286 (2412) |
|  | adl | vacuuming | 192 (80) | 42 (37) | 59 (34) | 3120 (1558) |
|  | adl | working at PC | 9 (18) | 7 (7) | 3 (7) | 21 (248) |
|  | stairs | slope up slope down | 467 (157) | 208 (107) | 308 (98) | 6119 (2076) |
|  | walking | walking 2.8 km/h | 347 (91) | 135 (55) | 184 (48) | 3974 (1048) |
|  | walking | walking 3.2 km/h | 384 (109) | 163 (64) | 216 (64) | 4049 (1206) |
|  | walking | walking 5.4 km/h | 665 (135) | 386 (122) | 478 (133) | 6191 (1136) |
|  | jogging | running 12 km/h | 1626 (199) | 1319 (264) | 1108 (161) | 16930 (3454) |
|  | jogging | running 7.6 km/h | 1116 (226) | 729 (205) | 694 (128) | 10858 (2276) |
|  | cycling | Cycling | 259 (107) | 99 (65) | 158 (82) | 5634 (2961) |
| hip | lying | lying horizontal | 3 (2) | 6 (4) | 2 (1) | 0 (0) |
|  | lying | lying left | 6 (6) | 11 (8) | 2 (1) | 2 (41) |
|  | lying | lying prone | 9 (36) | 7 (11) | 4 (16) | 95 (822) |
|  | lying | lying right | 1 (1) | 5 (7) | 1 (0) | 0 (0) |
|  | lying | natural lying | 11 (34) | 9 (8) | 3 (9) | 109 (882) |
|  | sitting | natural sitting | 11 (21) | 13 (6) | 3 (5) | 47 (428) |
|  | sitting | reclining | 7 (10) | 11 (7) | 3 (3) | 9 (145) |
|  | sitting | sitting - crossed legs left | 8 (8) | 14 (6) | 3 (3) | 1 (19) |
|  | sitting | sitting - crossed legs right | 7 (7) | 12 (7) | 3 (2) | 0 (5) |
|  | sitting | sitting - leaned backward | 8 (9) | 14 (7) | 3 (3) | 1 (59) |
|  | sitting | sitting - leaned forward | 11 (14) | 14 (7) | 4 (4) | 16 (188) |
|  | standing | standing still | 9 (9) | 14 (7) | 3 (3) | 5 (87) |
|  | standing | natural standing | 29 (25) | 16 (9) | 10 (11) | 85 (365) |
|  | standing | standing still - upper movement | 116 (61) | 34 (24) | 41 (28) | 721 (953) |
|  | adl | Windows cleaning | 103 (54) | 28 (16) | 33 (19) | 1080 (1216) |
|  | adl | Setting table | 99 (71) | 27 (20) | 30 (26) | 1297 (1810) |
|  | adl | get dressed | 125 (92) | 35 (30) | 48 (37) | 1678 (2443) |
|  | adl | hang out the laundry | 134 (102) | 32 (25) | 41 (31) | 2170 (3072) |
|  | adl | light activity | 224 (62) | 77 (30) | 121 (39) | 2554 (1090) |
|  | adl | put clean sheets on the bed | 133 (93) | 37 (31) | 49 (41) | 1817 (2105) |
|  | adl | reading newspaper | 16 (21) | 15 (8) | 4 (5) | 61 (411) |
|  | adl | smartphone usage | 48 (81) | 21 (23) | 20 (37) | 563 (1532) |
|  | adl | tidy up | 98 (96) | 26 (23) | 31 (32) | 1526 (2641) |
|  | adl | vacuuming | 145 (55) | 37 (21) | 51 (25) | 2253 (1495) |
|  | adl | working at PC | 10 (13) | 14 (7) | 3 (2) | 15 (186) |
|  | stairs | slope up slope down | 353 (136) | 145 (78) | 254 (103) | 4944 (1975) |
|  | walking | walking 2.8 km/h | 232 (45) | 82 (25) | 133 (26) | 2444 (744) |
|  | walking | walking 3.2 km/h | 252 (61) | 91 (29) | 148 (37) | 2599 (910) |
|  | walking | walking 5.4 km/h | 428 (62) | 180 (34) | 298 (45) | 4553 (799) |
|  | jogging | running 12 km/h | 1239 (137) | 667 (106) | 886 (106) | 10282 (1927) |
|  | jogging | running 7.6 km/h | 879 (163) | 439 (107) | 669 (127) | 8198 (1622) |
|  | cycling | Cycling | 125 (53) | 51 (25) | 69 (29) | 1021 (1152) |
| chest | lying | lying horizontal | 6 (3) | 7 (6) | 4 (1) | 0 (0) |
|  | lying | lying left | 6 (8) | 13 (6) | 3 (2) | 7 (125) |
|  | lying | lying prone | 7 (45) | 10 (11) | 4 (16) | 131 (1094) |
|  | lying | lying right | 6 (7) | 4 (5) | 3 (2) | 6 (102) |
|  | lying | natural lying | 13 (35) | 9 (8) | 5 (8) | 108 (872) |
|  | sitting | natural sitting | 18 (30) | 18 (8) | 7 (7) | 105 (735) |
|  | sitting | reclining | 11 (15) | 19 (8) | 5 (5) | 22 (305) |
|  | sitting | sitting - crossed legs left | 14 (14) | 19 (7) | 7 (5) | 7 (69) |
|  | sitting | sitting - crossed legs right | 13 (13) | 19 (7) | 6 (5) | 6 (103) |
|  | sitting | sitting - leaned backward | 16 (16) | 20 (8) | 8 (7) | 15 (134) |
|  | sitting | sitting - leaned forward | 19 (20) | 16 (5) | 8 (6)) | 40 (339) |
|  | standing | standing still | 14 (13) | 19 (7) | 6 (4) | 17 (209) |
|  | standing | natural standing | 37 (31) | 21 (8) | 13 (10) | 197 (687) |
|  | standing | standing still - upper movement | 159 (70) | 49 (24) | 65 (31) | 1466 (1836) |
|  | adl | Windows cleaning | 118 (56) | 32 (14) | 37 (19) | 1686 (1732) |
|  | adl | Setting table | 125 (82) | 28 (17) | 33 (22) | 2253 (2741) |
|  | adl | get dressed | 177 (112) | 41 (30) | 58 (36) | 3315 (3822) |
|  | adl | hang out the laundry | 179 (137) | 36 (27) | 47 (30) | 3698 (4872) |
|  | adl | light activity | 178 (42) | 70 (23) | 110 (32) | 1953 (884) |
|  | adl | put clean sheets on the bed | 167 (105) | 43 (28) | 56 (37) | 2980 (3177) |
|  | adl | reading newspaper | 28 (27) | 18 (6) | 9 (7) | 118 (540) |
|  | adl | smartphone usage | 50 (80) | 24 (20) | 21 (33) | 602 (1883) |
|  | adl | tidy up | 116 (103) | 25 (20) | 33 (28) | 1988 (3212) |
|  | adl | vacuuming | 150 (65) | 37 (16) | 46 (20) | 2774 (2191) |
|  | adl | working at PC | 17 (17) | 16 (5) | 7 (4) | 32 (284) |
|  | stairs | slope up slope down | 332 (127) | 146 (76) | 259 (102) | 5242 (2152) |
|  | walking | walking 2.8 km/h | 186 (37) | 76 (21) | 125 (26) | 1774 (783) |
|  | walking | walking 3.2 km/h | 200 (44) | 82 (24) | 136 (34) | 2035 (797) |
|  | walking | walking 5.4 km/h | 357 (64) | 155 (35) | 261 (53) | 4548 (1199) |
|  | jogging | running 12 km/h | 1204 (130) | 626 (108) | 945 (122) | 12029 (1852) |
|  | jogging | running 7.6 km/h | 863 (202) | 433 (123) | 713 (171) | 9369 (2009) |
|  | cycling | Cycling | 113 (53) | 50 (24) | 66 (28) | 441 (774) |
| ankle | lying | lying horizontal | 2 (5) | 5 (7) | 1 (1) | 5 (98) |
|  | lying | lying left | 5 (13) | 21 (15) | 2 (6) | 9 (78) |
|  | lying | lying prone | 10 (53) | 16 (25) | 5 (28) | 142 (1022) |
|  | lying | lying right | 1 (5) | 6 (9) | 1 (3) | 1 (26) |
|  | lying | natural lying | 13 (51) | 11 (16) | 5 (16) | 179 (1102) |
|  | sitting | natural sitting | 17 (54) | 8 (20) | 7 (23) | 161 (1059) |
|  | sitting | reclining | 6 (17) | 4 (6) | 3 (6) | 24 (197) |
|  | sitting | sitting - crossed legs left | 4 (11) | 4 (6) | 2 (3) | 1 (27) |
|  | sitting | sitting - crossed legs right | 8 (15) | 4 (7) | 3 (4) | 24 (280) |
|  | sitting | sitting - leaned backward | 10 (28) | 5 (12) | 4 (10) | 41 (358) |
|  | sitting | sitting - leaned forward | 13 (35) | 7 (14) | 5 (14) | 45 (459) |
|  | standing | standing still | 8 (13) | 5 (7) | 2 (2) | 20 (204) |
|  | standing | natural standing | 38 (45) | 8 (12) | 7 (10) | 364 (888) |
|  | standing | standing still - upper movement | 47 (38) | 14 (16) | 19 (15) | 228 (611) |
|  | adl | Windows cleaning | 144 (111) | 38 (56) | 52 (48) | 2075 (2051) |
|  | adl | Setting table | 124 (139) | 42 (77) | 57 (74) | 1636 (2536) |
|  | adl | get dressed | 155 (163) | 54 (89) | 77 (88) | 2045 (3035) |
|  | adl | hang out the laundry | 158 (147) | 55 (90) | 78 (82) | 2163 (2515) |
|  | adl | light activity | 456 (157) | 270 (133) | 324 (111) | 7672 (2897) |
|  | adl | put clean sheets on the bed | 176 (177) | 72 (115) | 96 (109) | 2464 (3118) |
|  | adl | reading newspaper | 15 (46) | 7 (18) | 6 (19) | 106 (734) |
|  | adl | smartphone usage | 85 (151) | 38 (78) | 47 (87) | 1322 (2688) |
|  | adl | tidy up | 97 (134) | 31 (73) | 40 (74) | 1293 (2507) |
|  | adl | vacuuming | 227 (124) | 77 (82) | 103 (69) | 3694 (2328) |
|  | adl | working at PC | 11 (31) | 5 (12) | 4 (12) | 53 (499) |
|  | stairs | slope up slope down | 678 (245) | 420 (217) | 525 (150) | 11479 (3768) |
|  | walking | walking 2.8 km/h | 481 (127) | 301 (110) | 371 (82) | 7241 (1734) |
|  | walking | walking 3.2 km/h | 528 (154) | 348 (130) | 422 (116) | 8046 (2252) |
|  | walking | walking 5.4 km/h | 869 (119) | 687 (129) | 740 (113) | 14887 (2296) |
|  | jogging | running 12 km/h | 1848 (218) | 1879 (351) | 1355 (157) | 36570 (4280) |
|  | jogging | running 7.6 km/h | 1217 (155) | 1034 (211) | 935 (111) | 24205 (3676) |
|  | cycling | Cycling | 387 (208) | 175 (123) | 258 (136) | 9728 (6037) |
| wrist | lying | lying horizontal | 3 (7) | 8 (8) | 2 (2) | 7 (124) |
|  | lying | lying left | 11 (36) | 12 (8) | 3 (6) | 110 (944) |
|  | lying | lying prone | 11 (71) | 10 (32) | 5 (31) | 189 (1531) |
|  | lying | lying right | 4 (12) | 3 (4) | 2 (2) | 12 (274) |
|  | lying | natural lying | 29 (90) | 10 (18) | 8 (22) | 472 (2284) |
|  | sitting | natural sitting | 38 (75) | 9 (16) | 13 (20) | 484 (1839) |
|  | sitting | reclining | 24 (60) | 7 (14) | 8 (16) | 285 (1386) |
|  | sitting | sitting - crossed legs left | 36 (72) | 9 (17) | 12 (21) | 453 (1768) |
|  | sitting | sitting - crossed legs right | 26 (66) | 8 (15) | 9 (17) | 299 (1577) |
|  | sitting | sitting - leaned backward | 46 (99) | 9 (20) | 14 (25) | 699 (2599) |
|  | sitting | sitting - leaned forward | 94 (109) | 19 (25) | 32 (32) | 1423 (2863) |
|  | standing | standing still | 18 (40) | 4 (11) | 6 (14) | 98 (1017) |
|  | standing | natural standing | 86 (116) | 16 (32) | 28 (37) | 1257 (3094) |
|  | standing | standing still - upper movement | 906 (438) | 472 (333) | 564 (263) | 17790 (8952) |
|  | adl | Windows cleaning | 570 (316) | 170 (157) | 246 (162) | 14083 (8657) |
|  | adl | Setting table | 365 (166) | 82 (58) | 132 (62) | 8323 (4781) |
|  | adl | get dressed | 565 (269) | 184 (153) | 245 (112) | 11513 (6625) |
|  | adl | hang out the laundry | 453 (196) | 113 (99) | 178 (82) | 10444 (5326) |
|  | adl | light activity | 234 (90) | 91 (47) | 135 (45) | 3838 (2641) |
|  | adl | put clean sheets on the bed | 602 (307) | 199 (196) | 266 (146) | 13140 (7334) |
|  | adl | reading newspaper | 130 (143) | 22 (31) | 36 (40) | 2562 (3897) |
|  | adl | smartphone usage | 73 (98) | 17 (32) | 33 (40) | 927 (2351) |
|  | adl | tidy up | 353 (185) | 92 (70) | 137 (69) | 7734 (5139) |
|  | adl | vacuuming | 324 (129) | 98 (60) | 126 (53) | 7299 (3762) |
|  | adl | working at PC | 56 (57) | 20 (16) | 26 (24) | 349 (1111) |
|  | stairs | slope up slope down | 398 (172) | 160 (93) | 253 (103) | 7469 (3849) |
|  | walking | walking 2.8 km/h | 239 (151) | 92 (82) | 140 (72) | 3795 (4027) |
|  | walking | walking 3.2 km/h | 258 (134) | 106 (64) | 151 (57) | 4287 (3867) |
|  | walking | walking 5.4 km/h | 359 (139) | 215 (108) | 220 (71) | 6863 (3754) |
|  | jogging | running 12 km/h | 1952 (308) | 1158 (301) | 1105 (192) | 29773 (6248) |
|  | jogging | running 7.6 km/h | 1224 (490) | 670 (254) | 775 (335) | 19194 (7020) |
|  | cycling | Cycling | 167 (100) | 80 (52) | 106 (46) | 1435 (2426) |
| upper arm | lying | lying left | 11 (21) | 22 (9) | 3 (4) | 47 (494) |
|  | lying | lying prone | 10 (56) | 12 (21) | 4 (20) | 151 (1255) |
|  | lying | lying right | 1 (3) | 3 (4) | 1 (1) | 1 (45) |
|  | lying | natural lying | 21 (65) | 10 (14) | 6 (15) | 303 (1628) |
|  | sitting | natural sitting | 24 (42) | 8 (10) | 9 (12) | 153 (837) |
|  | sitting | reclining | 14 (27) | 7 (8) | 6 (9) | 60 (521) |
|  | sitting | sitting - crossed legs left | 21 (30) | 9 (10) | 9 (12) | 79 (462) |
|  | sitting | sitting - crossed legs right | 18 (33) | 9 (9) | 7 (9) | 78 (693) |
|  | sitting | sitting - leaned backward | 26 (43) | 9 (11) | 9 (13) | 198 (942) |
|  | sitting | sitting - leaned forward | 40 (42) | 14 (10) | 12 (12) | 288 (885) |
|  | standing | standing still | 14 (18) | 7 (8) | 5 (4) | 28 (366) |
|  | standing | natural standing | 58 (63) | 15 (15) | 18 (18) | 562 (1514) |
|  | standing | standing still - upper movement | 466 (247) | 198 (165) | 258 (158) | 6345 (3934) |
|  | adl | Windows cleaning | 321 (161) | 78 (66) | 120 (75) | 6985 (4739) |
|  | adl | Setting table | 196 (86) | 46 (33) | 65 (31) | 3428 (2476) |
|  | adl | get dressed | 343 (171) | 94 (86) | 140 (75) | 6623 (4616) |
|  | adl | hang out the laundry | 264 (131) | 60 (55) | 93 (51) | 5550 (4084)) |
|  | adl | light activity | 210 (52) | 67 (24) | 110 (30) | 2711 (1337) |
|  | adl | put clean sheets on the bed | 340 (189) | 94 (86) | 131 (82) | 6451 (4783) |
|  | adl | reading newspaper | 62 (67) | 16 (16) | 18 (18) | 797 (1665) |
|  | adl | smartphone usage | 60 (89) | 19 (28) | 25 (39) | 753 (1938) |
|  | adl | tidy up | 204 (108) | 51 (40) | 73 (43) | 3572 (2962) |
|  | adl | vacuuming | 272 (106) | 65 (41) | 90 (46) | 6009 (3284) |
|  | adl | working at PC | 33 (36) | 13 (9) | 11 (10) | 173 (689) |
|  | stairs | slope up slope down | 370 (142) | 143 (82) | 247 (104) | 6206 (2441) |
|  | walking | walking 2.8 km/h | 210 (79) | 70 (42) | 117 (38) | 2515 (1819) |
|  | walking | walking 3.2 km/h | 222 (65) | 75 (27) | 124 (34) | 2693 (1675) |
|  | walking | walking 5.4 km/h | 315 (70) | 128 (34) | 206 (45) | 4083 (1709) |
|  | jogging | running 12 km/h | 1564 (175) | 899 (185) | 892 (123) | 23094 (3891) |
|  | jogging | running 7.6 km/h | 1023 (358 | 519 (219) | 634 (214) | 14903 (6100) |
|  | cycling | Cycling | 196 (69) | 74 (34) | 108 (36) | 1796 (1741) |
|  | lying | lying left | 11 (21) | 22 (9) | 3 (4) | 47 (494) |
