# Supplementary material for: Comparability of accelerometry outcomes across popular metrics and widespread sensor positions
Source: PLoS One. 2025 Dec 3;20(12):e0337897. doi: 10.1371/journal.pone.0337897 (PMC12674572; doi:10.1371/journal.pone.0337897)
Supplement: S3 File — (DOCX) [file pone.0337897.s003.docx]

**Supporting information 3 – Cutpoint analysis**

**Materials and Methods**

We identified metric- and sensor-location-specific thresholds for light physical activity (LPA), moderate physical activity (MPA) and vigorous physical activity (VPA) using receiver operating characteristic (ROC) curve analyses via the cutpointr package for R[1]. For this, we divided all 32 conditions into four physical activity intensity zones based on the corresponding MET values of the compendium of physical activity [2] and according to the commonly used MET thresholds proposed by Ainsworth et al.[2] Thus, activities were classified as LPA (>1.5 to <3 METs), MPA (≥3 to <6 METs), or VPA (≥6 METs). Using ROC analysis, we then identified cut points between the respective intensity zones that maximize the Youden’s index. The Youden’s index is calculated as sensitivity + specificity – 1 and thus maximizes sensitivity and specificity[3]. As a measure of accuracy, we provide the area under the curve (AUC) for each cut point.

**Results**

The results from the ROC analysis are shown in Table 3. For LPA, thresholds for wrist data were generally higher than thresholds of other locations, while thresholds for ankle data were higher than other location thresholds for MPA and VPA.

Metrics show similar mean AUCs (averaged across sensor locations and intensity zones), ranging from .81 (CPM) to .83 (MAI). Sensor locations also show similar mean AUCs (averaged across metrics and intensity zones), ranging from .79 (hip) to .84 (thigh).

LPA was most accurately distinguished from SED with hip-worn sensors and MAD data (AUC = .95). MPA was most accurately distinguished from LPA with thigh worn sensors and CPM data (AUC = .71). VPA was generally accurately distinguished from MPA with ankle, chest, hip, thigh and arm worn sensors across all metrics (AUC = .92 to .99).

**Table S3. metric- and location-specific cut points.**

| **Metric** | **Position** | **LPA** | | **MPA** | | **VPA** | |
| --- | --- | --- | --- | --- | --- | --- | --- |
|  |  | threshold | AUC | threshold | AUC | threshold | AUC |
| **MAI** | ankle | 15 | .93 | 704 | .66 | 999 | .99 |
|  | chest | 43 | .93 | 257 | .59 | 487 | >.99 |
|  | hip | 26 | .94 | 171 | .41 | 549 | >.99 |
|  | thigh | 21 | .94 | 505 | .64 | 743 | .99 |
|  | arm | 93 | .92 | 246 | .65 | 790 | .98 |
|  | wrist | 115 | .89 | 253 | .54 | 944 | .95 |
| **ENMO** | ankle | 38 | .83 | 498 | .63 | 783 | .99 |
|  | chest | 31 | .87 | 115 | .61 | 224 | >.99 |
|  | hip | 26 | .87 | 128 | .61 | 242 | >.99 |
|  | thigh | 25 | .84 | 238 | .63 | 418 | .99 |
|  | arm | 32 | .90 | 92 | .66 | 178 | .98 |
|  | wrist | 31 | .90 | 126 | .62 | 361 | .98 |
| **MAD** | ankle | 5 | .92 | 529 | .65 | 797 | .98 |
|  | chest | 13 | .94 | 84 | .40 | 380 | >.99 |
|  | hip | 7 | .95 | 91 | .39 | 379 | >.99 |
|  | thigh | 7 | .93 | 283 | .65 | 507 | .98 |
|  | arm | 35 | .94 | 151 | .65 | 273 | .98 |
|  | wrist | 64 | .92 | 172 | .58 | 544 | .96 |
| **CPM** | ankle | 60 | .82 | 10373 | .70 | 18279 | .99 |
|  | chest | 60 | .85 | 3143 | .60 | 6523 | .98 |
|  | hip | 60 | .84 | 3592 | .61 | 5706 | .98 |
|  | thigh | 60 | .85 | 5058 | .71 | 7902 | .98 |
|  | arm | 85 | .88 | 3413 | .62 | 13177 | .93 |
|  | wrist | 603 | .86 | 4202 | .53 | 15141 | .92 |

**Discussion**

To our knowledge, we are the first to estimate cut points for multiple metrics and sensor positions based on the same activity protocol, enabling a comparison between metrics and sensor positions. Cut-point estimations depend on many factors, such as the chosen wearable, the metric, the epoch length, the sensor position, the chosen activities and the demographics of the sample. Therefore, a wide range of cut points exists for different populations. The cut points we derived are within this range. For instance, our ENMO and MAD cut-points align well with previously published thresholds by Bakrania et al.[4] ENMO cut points for self-paced free-living walking (25.9 and 26.6 mg, depending on the sensor) compared well to our hip-worn LPA cut-point (26 mg). For the wrist, our ENMO LPA cut point (31 mg) falls within the range of light-intensity activities reported in their study (e.g. 25.8 mg for washing pots or 52.6 mg for sweeping the floor). As their cut-points were derived for individual activities rather than aggregated intensity categories, a direct comparison is difficult, yet the values are overall closely aligned. Similarly, our MAD-based LPA cut points are consistent with their findings, with 7 mg at the hip for LPA compared to their cut point of 8.5 mg for washing pots, and 64 mg at the wrist for LPA compared to their cut point of 66.1 or 67.1 mg for self-paced walking. These similarities support the plausibility and validity of our derived thresholds. Generally, our LPA cut points had good to excellent accuracy with AUCs ranging from .82 to .95. However, the MPA cut points we identified display poor to fair accuracy with AUCs ranging from .41 to .71. Cut points for VPA had excellent accuracy with AUCs ranging from .92 to .99. The overall smaller accuracy for the MPA thresholds could be due to the fact the activity protocol consisted of many activities of daily living (11 out of 32 activities) which are diverse, unstructured movements (e.g. putting sheets on the bed, window cleaning, getting dressed, vacuuming etc.). Since our LPA and MPA activities are mainly comprised of adl, a clear accelerometry-based distinction is very difficult. This illustrates how influential the choice of selected activities for accurate cut point calibration is and implies that single-sensor accelerometry-based intensity estimations are especially difficult for adl. This raises the question of which activities are useful for a valid calibration of cut-points and according to which criteria these activities should be selected. Despite numerous studies on cut-points, little attention has been paid to this question so far. Moreover, the cut-point approach has further limitations: For instance, identifying cut points is usually time-consuming and expensive, often leads to overfitting or collinearity between classes and is very population specific. Therefore, some researchers advise alternative approaches such as segmenting the data via unsupervised machine learning. Additionally, future machine learning approaches might enable more accurate energy expenditure estimation based on activity type recognition, which would make a rather simplistic cut-point approach obsolete.

Our results are limited by the fact that the cut points were identified based on the MET values of the compendium of physical activity. For more accuracy, cut points should be estimated based on energy measures such as indirect calorimetry, e.g, via cardiopulmonary exercise testing (CPET). For accurate cut point estimation, a free-living validation study is necessary.

**References**

1. Thiele C, Hirschfeld G. **cutpointr** : Improved Estimation and Validation of Optimal Cutpoints in *R*. J Stat Soft [Internet]. 2021 [cited 2025 Oct 10];98(11). Available from: http://www.jstatsoft.org/v98/i11/

2. Ainsworth BE, Haskell WL, Herrmann SD, Meckes N, Bassett DR, Tudor-Locke C, et al. 2011 Compendium of Physical Activities: a second update of codes and MET values. Med Sci Sports Exerc. 2011 Aug;43(8):1575–81.

3. Perkins NJ, Schisterman EF. The inconsistency of “optimal” cutpoints obtained using two criteria based on the receiver operating characteristic curve. Am J Epidemiol. 2006 Apr 1;163(7):670–5.

4. Bakrania K, Yates T, Rowlands AV, Esliger DW, Bunnewell S, Sanders J, et al. Intensity Thresholds on Raw Acceleration Data: Euclidean Norm Minus One (ENMO) and Mean Amplitude Deviation (MAD) Approaches. PLOS ONE. 2016;
